# Juvenile idiopathic arthritis in a premature baby: rare case report

**DOI:** 10.1093/omcr/omaa100

**Published:** 2020-11-24

**Authors:** Leen Jamel Doya, Fareeda Wasfy Bijow, Adnan Dayoub

**Affiliations:** Department of Pediatrics, Tishreen University Hospital, Lattakia, Syria

**Keywords:** Juvenile idiopathic arthritis, premature baby

## Abstract

Juvenile idiopathic arthritis (JIA) is chronic arthritis in children and adolescents. It is clinically diagnosed, which includes children under the age of 16 with arthritis for at least six weeks. Cases younger than six months of age are extremely rare. Here we report a rare case in the literature about Juvenile idiopathic arthritis in a premature baby, presenting at 21 days of age. The diagnosis was made according to clinical symptoms, laboratory analyses and duration of disease.

## INTRODUCTION

Juvenile idiopathic arthritis (JIA) is a chronic form of arthritis of unknown etiology that manifests before the age of 16. The symptoms of JIA persist for more than 6 weeks. The diagnosis is made after excluding other diseases that cause arthritis [1]. There are seven different types of JIA include oligoarticular (up to 4 joints affected), either persistent or extended (more than 4 joints affected after the first 6 months); polyarticular (more than 4 joints affected) with two different types; systemic arthritis; enthesitis-related arthritis and psoriatic arthritis [2].

The incidence of JIA varies across populations. Studies in European and North American populations have shown an incidence rate ranging from 20 to 50 cases per 100,000 population, possibly due to genetic and epigenetic aspects of the disease. The incidence of JIA in developing countries is unknown [3]. About half of the patients with JIA have oligoarthritis [4].

## CASE REPORT

A female premature infant was born at 29 weeks gestational age by normal spontaneous vaginal delivery because of premature rupture of membranes (PROM) and vaginal bleeding three days before the delivery with a birth weight of 1325 g. She was the first child of a 16-years-old mother. There were no other significant obstetric and familial histories. After birth, the premature infant was admitted to the neonatal intensive care unit (NICU). The physical examination at admission was normal with prematurity signs and all her vital signs were within the normal average. Because of the suspicion of infection, antibiotics were initiated after performing laboratory tests and blood culture. On the fourth day of life, enteral feeding was started via a nasogastric tube. A cranial ultrasound (CUS) examination was performed which was normal.

**Figure 1:**
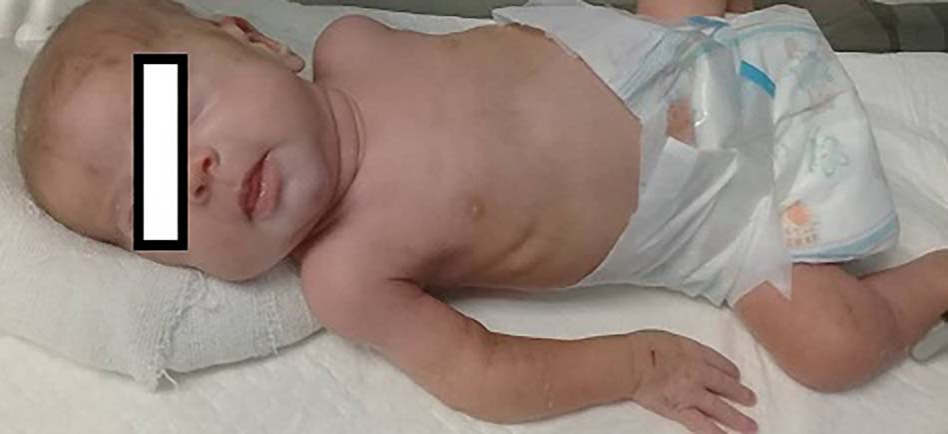
The right elbow joint with redness and swelling

On day 21 of life, redness and swelling appeared in the right elbow joint, with a circumference that exceeds the left by 2.5 cm with the absence of heat, and extra-articular symptoms (Fig. 1). The peripheral pulse was palpable and other extremities were normal. Laboratory results showed an elevation in inflammatory markers C- reactive protein (CRP), erythrocyte sedimentation rate (ESR), and reduction in hemoglobin (Hb 7.5 mg/dl), total protein and albumin. X-Ray of the right elbow was performed which showed abduction between right Humerus and Forearm bones with edema in subcutaneous connective tissue. The right elbow Ultrasound demonstrated no effusion. The patient was treated with antibiotics for two weeks without any improvement.

**Figure 2:**
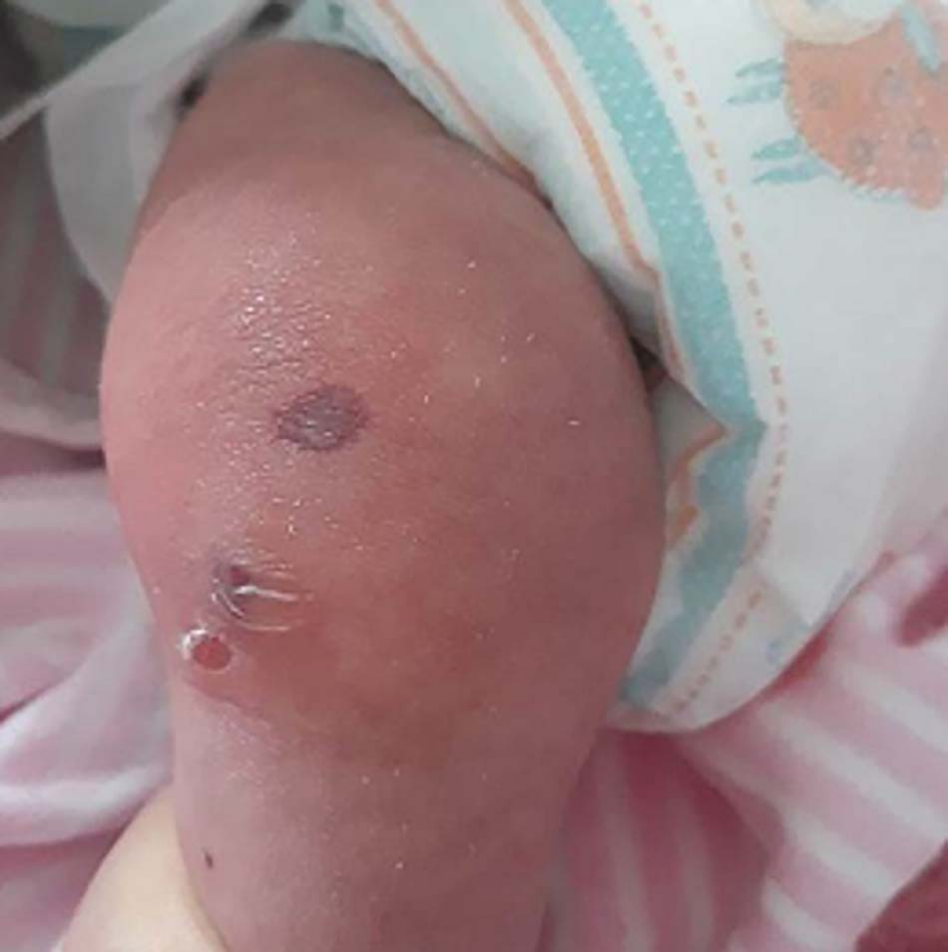
The swollen and redness right knee joint

On day 41, she developed swelling, redness, local heat, and slight pain on the mobilization of the right knee joint with limitation of movement (Fig. 2). The inflammatory markers were elevated with negative blood and Joint fluid culture that excluded the septic cause. A hematologic consultation was performed to exclude blood diseases and malignancies. The blood smear showed microcytic hypochromic anemia. Hemoglobin electrophoresis was normal, direct and indirect coombs tests were negative. Rheumatoid factor (RF), Anti-nuclear antibody (ANA), anti-neutrophil cytoplasmic antibodies (ANCA), anti-cyclic citrullinated peptide (anti-CCP) were negative. Serological Immunoglobulin was within the normal range (Table 1).

**Table 1:** The laboratory data of the case

Test	Result	Test	Result
White blood cells × (10^3^/μl)	8.5	ANA^a^ (U/ml)	0.4
Neutrophils (%)	44	ANCA^b^(U/ml)	10
Lymphocyte (%)	55	Anti-CCP^c^	1
Haemoglobin (g/dl)	7.5	RF^d^ (IU/ml)	4
Platelets × (10^3^/μl)	300	Direct coombs	Negative
Urea (mg/dl)	30	Indirect coombs	Negative
Creatinine (mg/dl)	0.5	IgG^e^(g/l)	10
Hemoglobin electrophoresis	Normal	IgM^f^(g/l)	0.20
ESR (1 h)	65	IgA^g^(g/l)	0.03
C-reactive protein (mg/dl)	50		

^a^Antinuclear antibody: <0.8 is interpreted as negative.

^b^Antineutrophil cytoplasmic autoantibody: <19 is interpreted as negative.

^c^Anti-cyclic citrullinated peptide antibody: <2 is interpreted as negative.

^d^Rheumatoid factor: <10 is interpreted as negative.

^e^Immunoglobulin G: normal range for age (6.5–16).

^f^Immunoglobulin M: normal value for age <0.25.

^g^Immunoglobulin A: normal range for age (0.01–0.04).

ESR, Erythrocyte sedimentation rate.

We performed echocardiography and a Funduscopic examination which were normal. The whole-body magnetic resonance imaging (WB-MRI) was normal. We were not able to perform a pet scan because of the lack of availability in our hospital.

On day 57, inflammatory signs appeared in the left knee joint as swelling in other extremities improved. Considering the clinical, serological, and radiology findings (pattern and duration of symptoms, migratory arthritis, RF negative, normal WB-MRI) and according to JIA classification, oligoarthritis on day 64 was diagnosed and treated with prednisolone. All of the clinical and laboratory signs were improved and the patient was discharged after 10 days.

We followed the patient for one year. During this year, the patient had arthritis only once at the age of 7 months. It was responding to Non-steroidal anti-inflammatory drugs (NSAIDs) and lasted for 2 days. Currently, the child is in good condition, all signs of inflammation are disappeared, good weight gain with good development in movement (stand with support) and the lengths of limbs are within the normal range.

## DISCUSSION

Oligoarticular JIA is chronic arthritis begins before age 16 years with the highest frequency in girls aged 1–3 years [5]. According to our reviews of medical literature, there was only one case in Iran associated with oligoarticular JIA in a full-term 20-day-old girl [6]. Here, we presented a rare case of JIA in a premature baby with clinical manifestations during the neonatal period (21 days of age).

Oligoarticular JIA affects the lower extremities, with the most frequently involved joints being the knee (30–50% of cases), followed by the ankle [7]. Predictors for oligoarticular JIA have been identified as wrists and ankle arthritis and high ESR at onset [8]. The diagnosis of JIA is made by exclusion, when suspected, requires a complete clinical evaluation, including family and personal history and recent pathologic events [9]. In our case, the patient was afebrile with swelled large joints (the elbow and knee) and high ESR that suggested the Oligoarticular subtype of JIA after excluding other diseases.

JIA demonstrates a good response to disease-modifying anti-rheumatic drugs (DMARDs), particularly methotrexate (MTX), which are well-tolerated and commonly used [10]. Traditionally, NSAIDs have been the mainstay treatment for all forms of JIA. It cannot be used as monotherapy for more than 2 months in persistent active arthritis [9]. Glucocorticoid medications are indicated for life-threatening systemic disease or uncontrolled JIA, and as an intra-articular agent [9]**.**

Based on the diagnosis of our patient, treatment with prednisolone started. We could not use NSAIDs because of the early prematurity, which is a contraindication for less than six months old. Also, we are not able to use MTX due to its renal, hepatic and pulmonary toxicity. We used prednisolone because of its efficacy and safety in preterm infants. The patient successfully improved after treatment and was discharged from the hospital with a gradual reduction of prednisolone.

## CONCLUSION

Our case is unique because we describe a rare case report in the literature about JIA in a premature baby. Even though JIA is rare in neonates, the diagnosis of this disease should be suspected in patients with joint inflammation at any age. Early diagnosis and treatment will lead to good outcomes and avoidance of serious clinical sequelae.

## WRITTEN INFORMED

Consent was obtained from the patient’s parents for publication of this case report and any accompanying images. A copy of the written consent is available for review by the Editor.
